# Supplementary material for: Morphology and Molecular Phylogeny of the Genus Stigeoclonium (Chaetophorales, Chlorophyta) from China, Including Descriptions of the Pseudostigeoclonium gen. nov
Source: Plants (Basel). 2024 Mar 6;13(5):748. doi: 10.3390/plants13050748 (PMC10935110; doi:10.3390/plants13050748)
Supplement: Supplementary file 1 [file plants-13-00748-s001.zip › plants-2865800-supplementary.pdf]

Table S1. The newly obtained voucher number, taxon, collection information and their GenBank accession numbers of Chaetophorales used for 18S rDNA, ITS2 and tufA sequence analyses.

Voucher number	Taxon	Isolator, isolation data and collection information	GenBank accession number		
			18S rDNA	ITS2	*tuf*A
bmA10	*Stigeoclonium* sp.	B. W. Liu, 2016, Xizang, China, on the stones in a stream, freshwater.	MK250079	MN428038	MN699101
HB201714	*Stigeoclonium* sp.	B. W. Liu, 2017, Wuhan, Hubei province, China, on the sticks in a still water, freshwater.	OP236760	OP243338	OP263076
HB201512	*Chaetophoropsis polyrhizum*	B. W. Liu, 2015, Suizhou, Hubei province, China, on rocks in a trench, stagnant water, freshwater.	MH002620	MH002620	MW468502
HN201708	*Stigeoclonium* sp.	B. W. Liu, 2017, Hengyang, Hunan province, China, at the water pipe outlet, freshwater.	OP236759	OP243337	MW468531
HB201705	*Stigeoclonium* sp.	B. W. Liu, 2017, Wuhan, Hubei province, China, on floating leaves in East Lake, freshwater.	OP236758	OP243336	MW468522
JX201714	*Stigeoclonium* sp.	B. W. Liu, 2017, Nanchang, Jiangxi province, China, Nanchang University, on the stones in a stream, freshwater.	OP236769	OP243348	MW468541
JX201712	*Chaetophoropsis attenuata*	B. W. Liu, 2017, Nanchang, Jiangxi province, China, Nanchang University, on the stones in a stream, freshwater.	OP236768	OP243347	MW468499
JX201710	*Stigeoclonium lubricum*	B. W. Liu, 2017, Ruijin, Jiangxi province, China, on the fishing net in a river, freshwater.	OP236767	OP243346	MW468525
HB201502	*Chaetophoropsis polyrhizum*	B. W. Liu, 2015, Wuhan, Hubei province, China, Wuhan Botanical Garden, Chinese Academy of Sciences, on water grasses in a pool, freshwater.	MH002619	MH002619	MW468501
JX201707	*Stigeoclonium amoenum*	B. W. Liu, 2017, Ruijin, Jiangxi province, China, on the stonges in a river, freshwater.	OP236767	OP243345	MW468515
JX201701	*Stigeoclonium amoenum*	B. W. Liu, 2017, Huichang, Jiangxi province, China, on water grasses in a river, freshwater.	OP236765	OP243344	MW468512
GXGL201705	*Stigeoclonium variabile*	B. W. Liu, 2017, Guilin, Guangxi province, China, on the walls of water pipes in a hot spring well, freshwater.	OP236745	OP243324	MW468579

HB201649	*Stigeoclonium* sp.	B. W. Liu, 2016, Wuhan, Hubei province, China, Wuhan Botanical Garden, Chinese Academy of Sciences, on a plastic bags in a pool, freshwater.	OP236757	OP243335	MW46853 0
HB201646	*Chaetophoropsis polyrhizum*	B. W. Liu, 2016, Wuhan, Hubei province, China, Wuhan Botanical Garden, Chinese Academy of Sciences, on water grasses in a pool, freshwater.	MH002621	MH002621	MN701587
HB201639	*Stigeoclonium helveticum*	B. W. Liu, 2016, Wuhan, Hubei province, China, on a waterweed stems in the Yangtze River, freshwater.	OP236756	OP243353	MW46855 3
HB201638	*Stigeoclonium* sp.	B. W. Liu, 2016, Wuhan, Hubei province, China, Wuhan Botanical Garden, Chinese Academy of Sciences, on water grasses in a pool, freshwater.	OP236755	OP243334	MW46854 0
HB201636	*Chaetophoropsis* sp.	B. W. Liu, 2016, Wuhan, Hubei province, China, Wuhan Botanical Garden, Chinese Academy of Sciences, on the bamboo slices in a pool, freshwater.	OP236754	OP243333	MW46850 0
HB201635	*Stigeoclonium* sp.	B. W. Liu, 2016, Wuhan, Hubei province, China, Wuhan Botanical Garden, Chinese Academy of Sciences, on the stick in a pool, freshwater.	MK250081	MN428036	MN701166
HB201631	*Stigeoclonium* sp.	B. W. Liu, 2016, Wuhan, Hubei province, China, Wuhan Botanical Garden, Chinese Academy of Sciences, on the leaves in a pool, freshwater.	OP236753	OP243332	MW46851 4
YN201601	*Stigeoclonium* sp.	B. W. Liu, 2016, Yuxi, Yunnan province, China, on the rocks on the shore beaten by fine waves in the Fuxian Lake Scenic Area, freshwater.	OP236775	OP243354	MW46851 1
HB201627	*Stigeoclonium aestivale*	B. W. Liu, 2016, Wuhan, Hubei province, China, Wuhan Botanical Garden, Chinese Academy of Sciences, on the stick in a pool, freshwater.	OP236752	OP243331	MW46851 9
HB201811	*Stigeoclonium* sp.	B. W. Liu, 2018, Wuhan, Hubei province, China, Wuhan Botanical Garden, Chinese Academy of Sciences, on the stick in a pool,	OP236764	OP243343	MW46858 6

		freshwater.			
HB201617	*Stigeoclonium* sp.	B. W. Liu, 2016, Huanggang, Hubei province, China, on the rock in a stream, freshwater.	OP236751	OP243330	MW468527
HB201611	*Stigeoclonium* sp.	B. W. Liu, 2016, Wuhan, Hubei province, China, on the stick in a stream, freshwater.	OP236750	OP243329	OP263075
HB201644F	*Chaetophoropsis attenuata*	B. W. Liu, 2016, Wuhan, Hubei province, China, Wuhan Botanical Garden, Chinese Academy of Sciences, on the stick in a pool, freshwater.	MH002616	MH002626	MN701985
HB201809	*Stigeoclonium* sp.	B. W. Liu, 2018, Wuhan, Hubei province, China, Wuhan Botanical Garden, Chinese Academy of Sciences, at the bottom of running water tank, freshwater.	OP236763	OP243342	MW468585
WDLC201608	*Stigeoclonium helveticum*	B. W. Liu, 2016, Wudalianchi, Heilongjiang province, China, on the rock in a stream, freshwater.	OP247563	OP243350	MW468554
HB201602	*Stigeoclonium* sp.	B. W. Liu, 2016, Wuhan, Hubei province, China, on the rock of East Lake, freshwater.	OP236749	OP243328	MW468548
HB201601	*Stigeoclonium helveticum*	B. W. Liu, 2016, Wuhan, Hubei province, China, on leather shoes impacted by sewage, freshwater.	OP236748	OP243327	MW468550
HB201600	*Stigeoclonium* sp.	B. W. Liu, 2016, Xianning, Hubei province, China, on the stones impacted by sewage, freshwater.	OP236747	OP243326	OP263074
LY201701	*Uronema confervicolum*	B. W. Liu, 2017, Luoyang, Henan province, China, on water grasses in a river, freshwater.	MK250084	MN428033	MN701586
TB201650	*Stigeoclonium* sp.	B. W. Liu, 2016, Xizang, China, on the stones in a stream, freshwater.	OP236771	OP256564	OP263077
HB201735	*Stigeoclonium* sp.	B. W. Liu, 2017, Huanggang, Hubei province, China, on the stones in a ditch, freshwater.	OP247562	OP243341	MW468545
HB201730	*Stigeoclonium* sp.	B. W. Liu, 2016, Yichang, Hubei province, China, on the stones in a stream, freshwater.	OP236762	OP243340	MW468513
TB201638	*Stigeoclonium* sp.	B. W. Liu, 2016, Xizang, China, on the stones in a stream, freshwater.	OP236770	OP243349	MW468529

HB201721	*Stigeoclonium* sp.	B. W. Liu, 2017, Wuhan, Hubei province, China, Wuhan Botanical Garden, Chinese Academy of Sciences, on leaves of aquatic plants, still freshwater.	OP236761	OP243339	MW468534
HB201509F	*Stigeoclonium* sp.	B. W. Liu, 2015, Wuhan, Hubei province, China, on the rock of East Lake, freshwater.	OP236746	OP243325	OP263073
AES201708	*Chaetophoropsis aershanensis*	B. W. Liu, 2017, Aershan, Hinggan, Inner mongolia province, China, on the stones in Halaha river, freshwater.	MH002611	MH002622	OP263071
GD201802	*Stigeoclonium* sp.	B. W. Liu, 2018, Qingyuan, Guangdong province, China, on the rock in a stream, freshwater.	OP236743	OP243322	OP263072
GD201805	*Stigeoclonium* sp.	B. W. Liu, 2018, Guangzhou, Guangdong province, China, on the rock in a stream, freshwater.	OP236744	OP243323	MW468587
YJ201801	*Stigeoclonium* sp.	B. W. Liu, 2018, Yuanjiang, Hunan province, China, on the rock of Dongting Lake, freshwater.	OP236772	OP243351	MW468592
YJ201802F	*Stigeoclonium* sp.	B. W. Liu, 2018, Yuanjiang, Hunan province, China, on the rock of Dongting Lake, freshwater.	OP236773	OP243352	MW468593
YJ201806	*Stigeoclonium* sp.	B. W. Liu, 2018, Yuanjiang, Hunan province, China, on sanbar of Dongting Lake, on the moist soil, freshwater.	OP236774	-	MW468594
HB201823	*Fritschiella tuberosa*	B. W. Liu, 2018, Wuhan, Hubei province, China, sanbar of Yangtze River, on the moist soil.	MN428041	MN428042	MN701160
AES201713	*Draparnaldia mutabilis*	B. W. Liu, 2017, Aershan, Hinggan, Inner mongolia province, China, on the stones in Halaha river, freshwater.	MK250078	MN428039	MN659372
